# Case Report: Membranoproliferative Glomerulonephritis, a Rare Clinical Manifestation of Abernethy Malformation Type II

**DOI:** 10.3389/fped.2021.647364

**Published:** 2021-03-17

**Authors:** Xue He, Yueling Zhu, Haidong Fu, Chunyue Feng, Zhixia Liu, Weizhong Gu, Yanyan Jin, Binbin Yang, Huijun Shen

**Affiliations:** ^1^Department of Nephrology, National Clinical Research Center for Child Health, National Children's Regional Medical Center, The Children's Hospital, Zhejiang University School of Medicine, Hangzhou, China; ^2^Department of Traditional Chinese Medicine, National Clinical Research Center for Child Health, National Children's Regional Medical Center, The Children's Hospital, Zhejiang University School of Medicine, Hangzhou, China; ^3^Department of Pathology, National Clinical Research Center for Child Health, National Children's Regional Medical Center, The Children's Hospital, Zhejiang University School of Medicine, Hangzhou, China; ^4^Department of General Surgery, National Clinical Research Center for Child Health, National Children's Regional Medical Center, The Children's Hospital, Zhejiang University School of Medicine, Hangzhou, China

**Keywords:** Abernethy malformation, congenital extrahepatic portosystemic shunt, membranoproliferative glomerulonephritis, arachnoid cyst, focal nodular hyperplasia

## Abstract

This report describes an 8-year-old male who presented with clinical manifestations including systemic edema, heavy proteinuria, hypoproteinemia, and persistent hypocomplementemia. Arachnoid cysts and focal nodular hyperplasia were also detected. Imaging examination and renal biopsy were performed, and Abernethy malformation type II with immune complex-mediated membranoproliferative glomerulonephritis was considered the diagnosis. Due to the persistence of embryonic vessels, Abernethy malformation is a rare congenital vascular malformation of the splanchnic venous system, which can be classified as type I (end-to-side shunt) and type II (side-to-side shunt). Abernethy malformation with glomerulonephritis remains extremely rare. In the patient described, glomerulonephritis mediated by immune complex with “full-house” positive immunohistochemistry was confirmed on renal biopsy. In addition, he was treated with glucocorticoids and tacrolimus. Whether surgical treatment is necessary should be determined according to the state of the disease in the later stages. The present case reflects the association between the congenital portosystemic shunt and the renal region and, to the authors' knowledge, may be the first report to describe arachnoid cysts as a symptom of Abernethy malformation.

## Background

Abernethy malformation, first described by John Abernethy in 1973 (1), has also been known as congenital extrahepatic portosystemic shunt. According to a clinical imaging report by Kopec et al. (2) in 2016, it has been described in only 101 patients since 1973, with an estimated prevalence of between 1 per 30,000 to 1 per 50,000 cases (3). Caused by abnormal development of the umbilical vein and yolk vein in the embryo, it is regarded to be a rare anomaly involving the portal venous system, leading to abnormal shunt between the portal vein (PV) and inferior vena cava (IVC) (4). Through a complete or partial shunt, blood from the abdominal organs and intestinal system drains into the systemic circulation bypassing the liver. It is divided into two types according to the type of anastomosis between the PV and IVC, and the presence of intrahepatic PV supply. Type I Abernethy malformation is characterized by complete end-to-side portocaval shunt in relation to the congenital absence of portal venous, while the PV is partially discharged into the IVC via side-by-side anastomoses in type II (5). The clinical manifestations of Abernethy malformation are diverse and vary significantly. Some are associated with serious complications, such as gastrointestinal hemorrhage, hepatic encephalopathy, and liver tumors, while others are found only on physical examination without overt clinical manifestations (3, 6, 7). Generally, type I Abernethy malformation is significantly more severe than type II, at least in term of hepatic complications (7). Abernethy malformation with kidney disease is extremely rare. To our knowledge, there have been no more than five cases reported in the literature, although the clinical manifestations and pathological patterns were different (2, 8). The present article describes an 8-year-old boy exhibiting edema, heavy proteinuria, hypoproteinemia, persistent hypocomplementemia and arachnoid cysts. Based on analysis of clinical manifestations, and laboratory and imaging examinations, type II Abernethy malformation with immune complex-mediated membranoproliferative glomerulonephritis (MPGN) was considered the diagnosis.

## Case presentation

**Figure 1:**
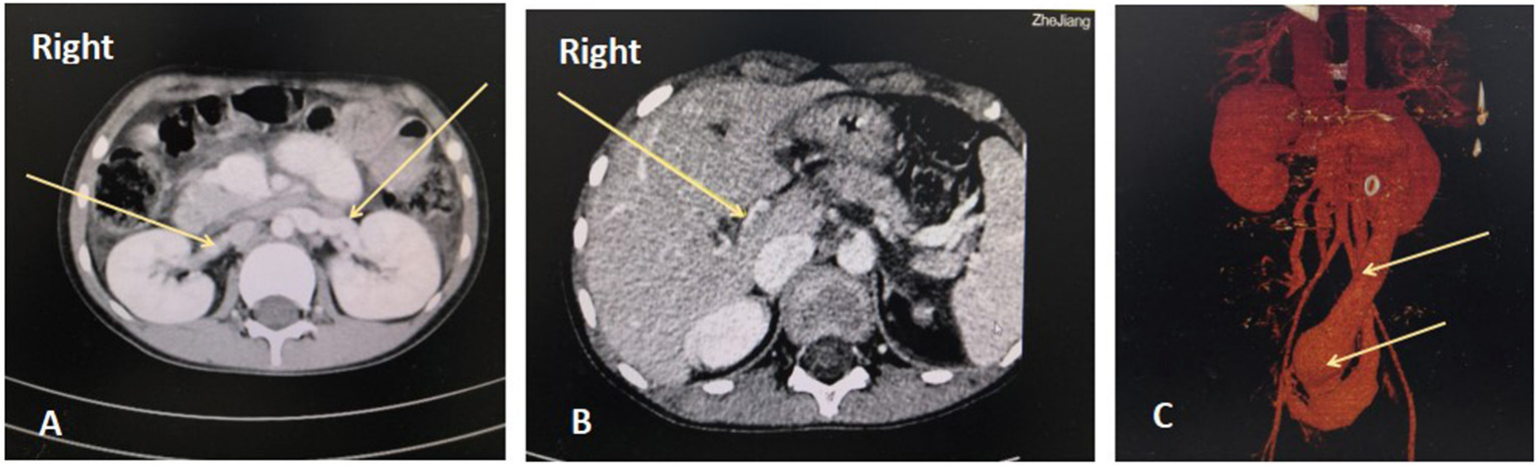
Abdominal enhanced computed tomography angiography (CTA). A. Bilateral renal veins are enlarged and thickened. B. The right branch of the portal vein is extremely thin, with a diameter of 1.2 mm. The left branch and the junction are not apparent in the images. C. The inferior vena cava and pelvic vein are remarkably dilated, and the widest point is 41 mm.

**Figure 2:**
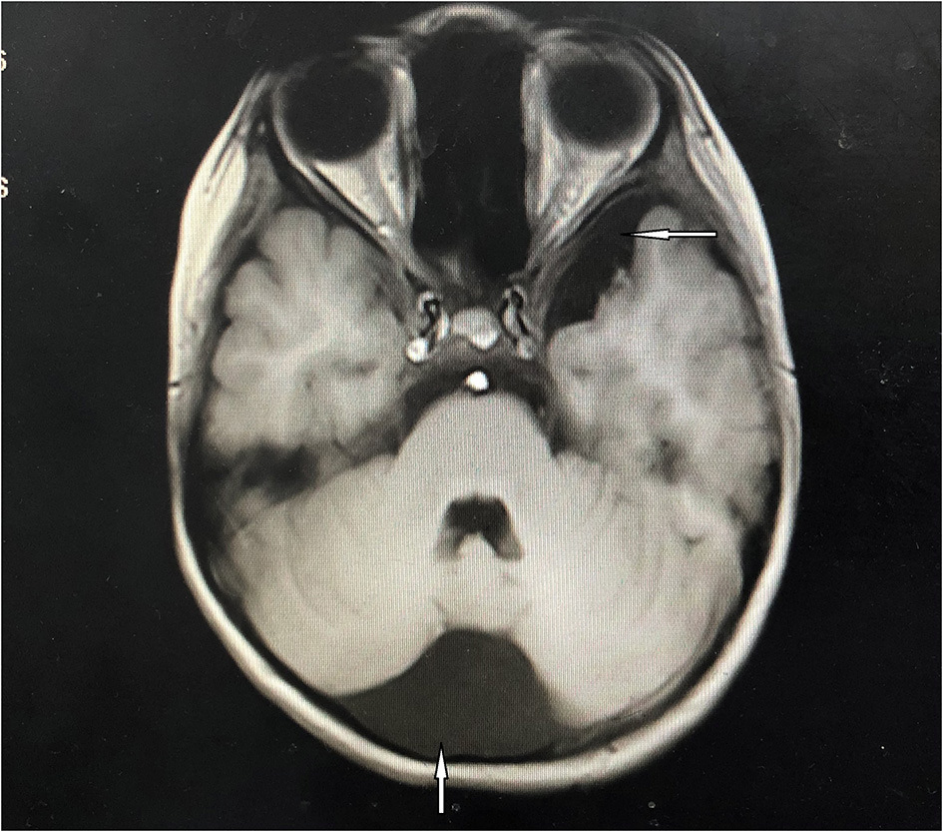
Cranial magnetic resonance imaging. Arachnoid cysts of occipital (the arrow underlying) and left middle cranial fossa (the arrow above) had been detected.

**Figure 3:**
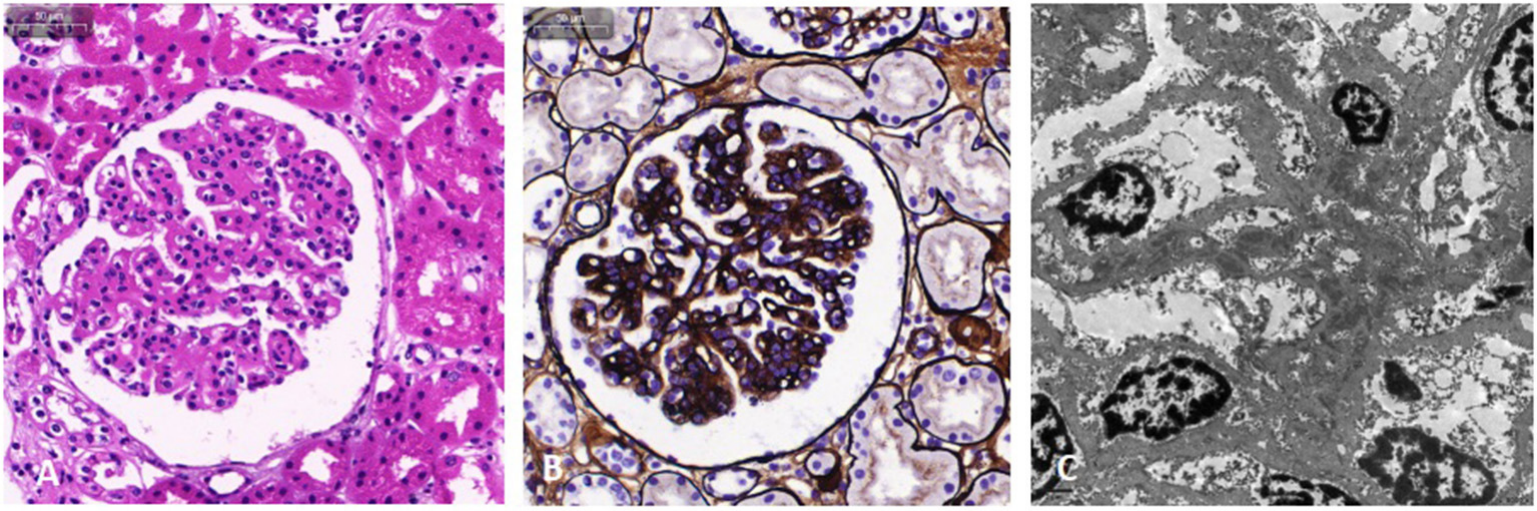
Light microscopy and electron microscopy images of kidney involvement. Hematoxylin and eosin stain **(A)** and Jones silver stain **(B)** are light microscopy images. The mesangial and endothelial cells exhibit diffuse proliferation. The capillaries are narrow, the capillary loop slightly thickened, and a “dual-track” sign can be partially observed. In addition, epithelial cells of the renal tubule exhibit granule denaturation while there was no obvious atrophy of renal tubules and no interstitial fibrosis. **(C)** Electron microscopy image. Electron dense deposits were observed in the mesangial and deputy mesangial regions, as well as a small amount of electron-dense deposit in the subepithelium.

The patient was an 8-year-old male, born at 40 weeks' gestation by normal vaginal delivery, with a weight of 3,200 g. He was the second child in the family, and his older sister and younger brother are healthy. In addition, his parents are healthy and unrelated, and his mother denied any history of taking teratogenic drugs, heavy drinking, or diabetes during pregnancy. Over the past 2 years, the boy occasionally exhibited periorbital edema. However, the current symptoms disappeared spontaneously within a short time and, as such, had not attracted much attention. In March 2020, he experienced sudden-onset systemic edema and his urine output decreased significantly within 5 days. Subsequently, he was hospitalized in the authors' department where a thorough physical examination was performed. Although his blood pressure was normal, he exhibited severe pitting edema in his legs, his eyelids and scrotum became extremely swollen, and his abdomen also began to swell without the subcutaneous varicose vein of the abdominal wall. Urinary examination revealed a 24 h urinary protein value of 1.2–6 g (46–230 mg/m^2^/h). Hematology revealed 100–200 red blood cells per high-powered field, and random routine urine examination revealed a urine protein level of 2+ to 4+. Laboratory investigations yielded the following findings: albumin, 17.3 g/L; cholesterol, 7.48 mmol/L; blood ammonia, 73 μmol/L; erythrocyte sedimentation rate, 4~11 mm/h; complement C3, 0.26~0.65 g/L; ceruloplasmin, 0.16 g/L; plasma fibrinogen, 0.72~1.27 g/L; D-dimer, 4.1~17.5 mg/L; serum creatinine value, 39–42 μmol/L; and the corresponding estimated glomerular filtration rate was normal. Antinuclear antibody titer was 1:80, and antineutrophil cytoplasmic antibody remained negative. Complete blood count, blood gas, blood culture, alanine aminotransferase, aspartate aminotransferase, total bilirubin and gamma-glutamyl transferase were normal. Moreover, thyroid function, serum parathyroid hormone and tumor markers were also normal. Serological tests for viral hepatitis and infectious criteria were negative. Laboratory findings on admission are summarized in Table 1. Heart Doppler ultrasound was normal. Abdominal ultrasonography (US) revealed that the left liver had lost its normal shape, and a low echogenic area, measuring 9.2 cm × 8.3 cm × 5.4 cm, could also be observed in the left lobe and right anterior lobe of the liver. Focal nodular hyperplasia (FNH) was considered. In addition, the spleen was enlarged, with its lower edge 1 cm below the costal margin. Abdominal vascular US revealed that the intrahepatic PV was poorly developed and the PV communicated with the left internal iliac vein. As revealed on abdominal enhanced computed tomography angiography (CTA), the intrahepatic PV was extremely thin, the external hepatic PV communicated with the body vein, and the pelvic vein was significantly dilated (Figure 1). Abernethy malformation type II was confirmed on the imaging examinations. Renal US indicated that the left kidney was 10.8 × 4.7 cm while the right kidney was 10.7 × 4.7 cm in size; both kidneys were plump, with enhanced cortical echo. Arachnoid cysts of the occipital (5.69 × 2.58 cm) and left middle cranial fossa (3.85 × 1.03 cm) were detected on abdominal enhanced computed (MRI), while patchy abnormal signal shadows were apparent on both sides of the globus pallidus and cerebral peduncle (Figure 2). Considering the possibility of early stage hepatic encephalopathy, electroencephalogram (EEG) was performed and an intelligence quotient test was administered. EEG was normal while the Wechsler Intelligence Scale indicated an intelligence quotient score of only 79. However, physical examination of the nervous system revealed no abnormalities. Subsequently, kidney biopsy was performed. Under optical microscopy, mesangial cells and endothelial cells exhibited diffuse proliferation, the capillaries were narrow, the capillary loop was slightly thickened, and a “dual-track” sign could be partially observed (Figures 3A,B). According to immunofluorescence results, immunoglobulin (Ig) G 2+, IgA 2+, IgM +, C3 2+, C1q +, and C4 + were irregularly deposited at the capillary loops and mesangial regions. Electron microscopy confirmed that the segmental mesangial insertion could be observed in the glomerular basement membrane, with proliferation of mesangial cells and stroma. Simultaneously, electron-dense deposits were observed in mesangial and deputy mesangial regions, as well as a small amount of electron-dense deposit in the subepithelium (Figure 3C). Glomerulonephritis mediated by immune complex was confirmed. Whole-exome sequencing did not reveal highly disease-related variants. Based on clinical manifestations, laboratory examination, and pathological analysis, the patient was diagnosed with type II Abernethy malformation with MPGN.

**Table 1:** Laboratory data on admission.

**Urinary examination**	**Value**	**Normal value**
24 h urinary protein quantitation	1.2~6g/24h	<0.5 g/24 h
Urine protein	2+~4+	-
UACR	2.13~8.56 mg/mg Cr	<0.2 mg/mg Cr
Urine erythrocyte	67–200/HP	0–3/HP
Urine culture	negative	negative
**Hematology**		
WBC	5.5 × 10^9^/L	4–12 × 10^9^/L
RBC	4.07 × 10^12^/L	3.5–5.5 × 10^12^/L
Hb	120 g/L	110–150 g/L
PLT	180 × 10^9^/L	100–400 × 10^9^/L
**Coagulation**		
PT	12.4 s	9–14 s
APTT	26.3 s	13–23 s
FIB	1.27 g/L	1.8–4 g/L
D—dimer	4.12 mg/L	<0.55 mg/L
**Blood chemistry**		
Alb	17.3 g/L	32–52 g/L
ALT	20 U/L	<50U/L
AST	54 U/L	15–60U/L
γ-GGT	11 U/L	8–57 U/L
CHOL	7.48 mmol/L	3–5.7 mmol/L
TBA	76.1 μmol/L	0–12 μmol/L
LDH	304U/L	110–290 U/L
Cr	39 μmol/L	15–77 μmol/L
Complement C3	0.262 g/L	0.9–1.8 g/L
Complement C4	0.039 g/L	0.1–0.4 g/L
Blood culture	negative	negative
ANA	1:80	negative
ANCA	negative	negative
ESR	11 mm/h	0–20 mm/h
Blood ammonia	73 μmol/L	18–72 μmol/L

*UACR, urinary albumin to creatinine ratio; WBC, white blood cell; RBC, red blood cell; Hb, hemoglobin; PLT, platelets; PT, prothrombin time; APTT, activated partial thromboplastin time; FIB, fibrinogen; Alb, albumin; ALT, alanine transaminase; AST, aspartate transaminase; γ-GGT, gamma-glutamyl transferase; CHOL, cholesterol; TBA, total bile acid; LDH, lactate dehydrogenase; Cr, creatinine; ESR, erythrocyte sedimentation rate*.

Currently, there is no unified nor clear understanding for the treatment of Abernethy malformation. The presence of kidney disorder significantly increases the risk for surgery. An expert panel discussion was held and drug treatment for kidney disorder was also recommended. Whether surgical treatment is necessary should be determined according to the state of the disease in the later stages. The patient was first treated with prednisone (2 mg/kg/day) for 4 weeks. However, urinary protein was maintained at 2+ to 3+. Subsequently, he was administered two courses of intravenous pulses of high-dose methylprednisolone (0.5 g/day). Each course lasted for 3 days and administered at an interval of 1 week. In addition, oral prednisone was continued between the two courses. Edema had subsided significantly and complement C3 level increased from 0.26 g/L to 0.65 g/L. However, the urinary protein did not turn negative. Subsequently, tacrolimus (1 mg/time, twice per day) and prednisone (1 mg/kg/day) were administered. In addition to glucocorticoids and tacrolimus, he also underwent plasma infusion to correct coagulation dysfunction. Other drugs for symptomatic treatment contain diuretics (furosemide and spironolactone) and anticoagulants (low molecular weight heparin and dipyridamole). Fortunately, urine protein turned negative after approximately 8 weeks' treatment. He was treated with prednisone and tacrolimus every day and checked regularly. The dosage of medication was gradually tapered. Urinary protein was maintained at – to +.

## Discussion and Conclusions

The clinical manifestations of Abernethy malformation are diverse, and can be summarized as the follows. First, other congenital malformations associated with Abernethy malformation. Among reported cases, > 22% of patients had congenital heart disease (CHD) (9). Other anomalies include abnormalities of the spleen, urinary and male genital tract, and brain and skeletal anomalies (9, 10). Associated malformations always remain common in type I Abernethy malformation, which are less frequent in type II (4). Second, hepatic dysfunction, nodular hyperplasia, or tumor are caused by inadequate PV perfusion of the liver. Nodular liver lesions were observed in approximately one-half of reported cases (11, 12). Most of the lesions, such as FNH (36.73%), were benign. In addition, other reported lesions include nodular regenerated hyperplasia (16.33%), hepatoblastoma (4.08%), hepatic adenoma (10.20%), hepatocellular carcinoma (26.53%) and cirrhosis (6.12%) (6, 13). Third, due to portacaval shunt of Abernethy malformation, extrahepatic manifestations include hepatic encephalopathy, pulmonary hypertension, hepatopulmonary syndrome, and glomerulonephritis (8, 14–16). Fourth, gastrointestinal hemorrhage and subcutaneous varicose veins of the abdominal wall which are caused by the obstruction of blood in the esophageal, periumbilical, and rectal venous plexuses (3, 17).

With advances in of imaging technology, the number of reported cases of Abernethy malformation has increased in recent years. Abernethy malformation can be confirmed using B-mode US, CT, multi-slice CT and digital substraction angiography (18). Regarding our patient, type II Abernethy malformation was confirmed using abdominal vascular US and CTA. Moreover, he did not have congenital heart disease, pulmonary hypertension, hepatopulmonary syndrome, or experience gastrointestinal hemorrhage, which is consistent with the literature reports of Abernethy malformation type II. Nevertheless, arachnoid cysts in the occipital and left middle cranial fossa were detected using cranial MRI, despite the patient not having a history of intracranial infection. As a result, it is conceivable that abnormal development of the early embryo may result in Abernethy malformation and arachnoid cysts simultaneously. As an associated symptom of Abernethy malformation, arachnoid cyst(s) may be primarily reported. In addition, blood ammonia level was 73 μmol/L, and patchy abnormal signal shadows could be observed on both sides of the globus pallidus and cerebral peduncle on cranial MRI, reflecting the early stage of hepatic encephalopathy. A low echogenic area in the liver was observed using B-mode US, and FNH should be primarily considered.

Abernethy malformation with glomerulonephritis remains extremely rare. To our knowledge, there have been no more than five cases reported in the literatures (2, 8, 19, 20). The main clinical manifestation is albuminuria, which may be accompanied by hematuria and abnormal renal function. In accordance with the current literature, pathological patterns exhibit MPGN (IgA deposition predominates as indicated by immunofluorescence), IgA nephropathy, and membranous nephropathy (8, 19, 20). Our patient exhibited edema, heavy albuminuria, hematuria, hypoproteinemia, and persistent hypocomplementemia. In our patient, MPGN was considered as the diagnosis. At the same time, glomerulonephritis mediated by immune complex with “full house” positive immunohistochemistry was confirmed on renal biopsy. It was acknowledged that immune complex-mediated MPGN with full-house immunohistochemistry had not been reported in cases of Abernethy malformation. The term “membranoproliferative glomerulonephritis” is used to describe a specific histological form of glomerulonephritis characterized by diffuse mesangial hypercellularity, endocapillary proliferation, thickening of the capillary wall, lobulation of the glomerular tuft, as well as the split of the glomerular capillary wall. Based on its pathogenesis, it has been classified into immune complex-mediated and complement-mediated MPGN and, moreover, can be subdivided into idiopathic and secondary forms. The immune complex-mediated type is secondary to chronic infections, autoimmune diseases, or monoclonal gammopathy (21, 22). Recently, the presence of a portosystemic shunt has been reported to be in association with MPGN. Smet et al. described a patient who had constructed a side-to-side portocaval shunt for two decades and subsequently experienced type I MPGN (23). Soma et al. described three patients who developed MPGN seven to 13 years after the construction of a portosystemic shunt for non-cirrhotic portal hypertension (24). Consequently, functional bypass of the hepatic reticuloendothelial system by the portosystemic shunt reduced the clearance of immune complexes, which was considered to be a critical role (23). In addition, Schaeffer et al. described a patient who was simultaneously diagnosed with Abernethy malformation type II and IgA glomerulonephritis, pulmonary hypertension, and multiple liver tumors. The authors also proposed a pathogenetic basis for the current multisystemic presentation including release into the systemic circulation of unfiltered bacteria, vasoactive substances, and IgA-antigen complexes (20). Our patient may have had e a special type of immune complex-mediated secondary MPGN. Therefore, we propose two mechanisms that could lead to kidney disorder. First is the bypassing and consequent inability of the liver to clear circulating IgA immune complexes that may originate from the intestinal mucosa and end up being filtered by the renal glomeruli, thus causing deposition and subsequent pattern of kidney injury. Another postulated cause is bacteremia resulting from impaired liver clearance of bacteria. Subsequently, the chronic infection can lead to post-infectious glomerulonephritis.

Currently, there is no unified nor clear understanding for the treatment of Abernethy malformation. Treatment can be determined according to disease type, condition of the shunt, and the individual situation of the patient. Type I is prone to lead to cirrhosis and liver cancer caused by the absence of portal vein as well as continuous insufficient blood supply to the liver. Consequently, liver transplantation is considered to be the main radical treatment method (12, 25, 26). In terms of type II, the opening of collateral circulation can relieve the pressure of PV, which may be conducive to the patient. Conservative treatment is selected in the absence of overt clinical symptoms. Closing parts of the collateral circulation and ligation of abnormal shunt vessels are considered, with excessive collateral circulation pressure, to be high risk for bleeding due to varicose veins, or intractable hepatic encephalopathy (16, 27–29). The number of cases similar to our patient reported globally remains limited. To date, there have been no reports in the literature describing whether closing the shunt can improve nephrotic symptoms. Meanwhile, the presence of kidney disorder significantly increases the risk for surgery. Albuminuria was controlled in the short term by drug treatment while the long-term prognosis is not favorable. In the future, the patient may develop malignant lesions of the liver, severe gastrointestinal bleeding, and chronic renal failure. The patient should be followed up and undergo long-term observation. Whether surgical treatment is necessary should be determined according to the state of the disease in the later stages. To date, the patient and his parents are compliant with the treatment, optimistic, and follow-up regularly.

In conclusion, accompanied by MPGN and arachnoid cysts, we described a patient who exhibited Abernethy malformation. MPGN with “full house” positive immunohistochemical immunohistochemistry is extremely rare in Abernethy malformation. Our cases highlighted the association between congenital portosystemic shunt and the renal region. Moreover, arachnoid cysts may be primarily reported as a congenital malformation in association with Abernethy malformation.

## Data Availability Statement

The raw data supporting the conclusions of this article will be made available by the authors, without undue reservation.

## Ethics Statement

This study was approved by the Ethical Review Board of The Children's Hospital of Zhejiang University School of Medicine. Written informed consent to participate in this study was provided by the participants' legal guardian/next of kin.

## Author Contributions

XH, YZ, and HS analyzed the data and wrote the manuscript. HF, WG, XH, ZL, CF, YJ, and BY contributed to clinical and instrumental data. All authors read and approved the final manuscript.

## Conflict of Interest

The authors declare that the research was conducted in the absence of any commercial or financial relationships that could be construed as a potential conflict of interest.

## Abbreviations

MPGN: membranoproliferative glomerulonephritis
PV: portal vein
IVC: inferior vena cava
FNH: Focal nodular hyperplasia
CTA: computed tomography angiography.
